# Neural Correlates of Spatial Navigation in Primate Hippocampus

**DOI:** 10.1007/s12264-022-00968-w

**Published:** 2022-11-02

**Authors:** Dun Mao

**Affiliations:** 1 Center for Excellence in Brain Science and Intelligent Technology, Institute of Neuroscience, Chinese Academy of Sciences, Shanghai, 200031 China; 2 University of Chinese Academy of Sciences, Beijing, 100049 China

**Keywords:** Spatial navigation, Primate, Hippocampus, Theta activity, Mixed selectivity, Task dependency, Eye and head coordinates

## Abstract

The hippocampus has been extensively implicated in spatial navigation in rodents and more recently in bats. Numerous studies have revealed that various kinds of spatial information are encoded across hippocampal regions. In contrast, investigations of spatial behavioral correlates in the primate hippocampus are scarce and have been mostly limited to head-restrained subjects during virtual navigation. However, recent advances made in freely-moving primates suggest marked differences in spatial representations from rodents, albeit some similarities. Here, we review empirical studies examining the neural correlates of spatial navigation in the primate (including human) hippocampus at the levels of local field potentials and single units. The lower frequency theta oscillations are often intermittent. Single neuron responses are highly mixed and task-dependent. We also discuss neuronal selectivity in the eye and head coordinates. Finally, we propose that future studies should focus on investigating both intrinsic and extrinsic population activity and examining spatial coding properties in large-scale hippocampal-neocortical networks across tasks.

## Introduction

The hippocampus integrates multiple sources of external and internal information, including sensory inputs, ongoing tasks, self-motion, and intrinsic dynamics. The exact role of the hippocampus in various cognitive tasks is debated, but the two most well-accepted functions pertain to spatial navigation and memory, which may fit into a common framework as a cognitive relational map [1–6].

Lesion studies have established the causal involvement of the rodent hippocampus in spatial learning and memory in maze tasks, in which animals need to locate themselves using environmental landmarks [7, 8]. Neural correlates of spatial navigation have been extensively studied in rodents, starting from the discovery of hippocampal place cells that become active at specific locations in the environment [9]. Since then, the characteristics of place cells have been thoroughly investigated [10–18], including their three-dimensional properties, that also extend to the bat hippocampus [19, 20]. More recent studies also demonstrate the causal effect of place cell activity on goal-directed locomotor behavior in a virtual linear track [21]. Place cells coexist with grid cells and head direction cells found in adjacent structures: Grid cells fire at regular, hexagonal locations; head direction cells activate when the animal’s head points in certain directions [22–25]. The spatial selectivity of these cells also extends to three dimensions, although the properties along the vertical axis may be different [26–28]. At the level of network activity, the prominent theta oscillations (5 Hz–10 Hz) show a strong correlation with locomotion in the rodent hippocampus [29], but much less so in the bat hippocampus, in which theta occurs in short bouts [25].

In contrast to rodents and bats, the primate hippocampus has scarcely been studied in a naturalistic context, but is largely restricted to immobile subjects during virtual navigation. The primate hippocampus often shows short-lived, lower frequency theta activity (3 Hz–4 Hz) during both virtual and real-world navigation [30–32]. At the level of single neurons, there tends to be an even larger discrepancy between rodents and primates. Although some neurons show rodent-like place fields in the virtual environment and real-world linear track [33, 34], this result dissipates during free navigation in an open arena [35]. Strikingly, many neurons show mixed selectivity to multiple spatial variables across hippocampal regions, with head orientation and eye movement properties being dominant [35]. Another provocative finding is that neurons exhibit selectivity to gaze location under either full or partial body restraint [36–38], which may imply another aspect of important differences between rodents and primates.

In this review, we discuss findings in the primate hippocampus during virtual and real-world navigation, with occasional comparisons to rodents and bats. We first summarize recent findings by focusing on the theta band activity and its correlation with behavior. We then review the findings on spatial selectivity at the single-cell level, emphasizing task-dependent, multiplexed responses. In the end, we propose a few open questions that future studies should address.

## Theta Activity and Theta Sequences in Rodents and Bats

**Fig. 1:**
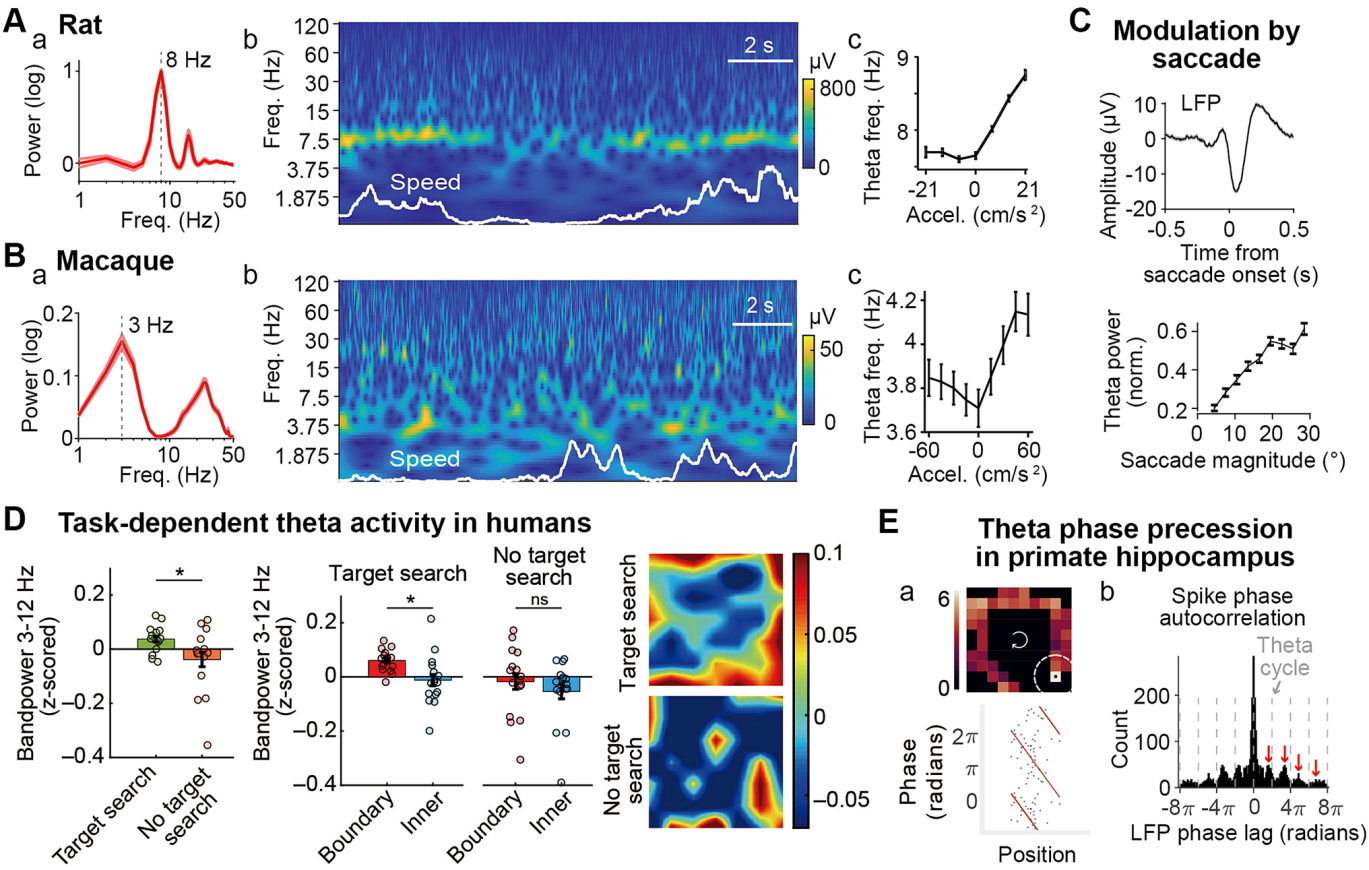
Hippocampal theta activity across species. **Aa** Power spectrum of the oscillatory component of the rat hippocampal LFP during free foraging. Shaded area indicates SEM across 3 animals. Freq., frequency. **Ab** Scalogram of a short segment of rat hippocampal LFP using wavelet analysis. Speed is shown as the white trace. **Ac** Hippocampal theta frequency as a function of acceleration (Accel.). **B** As in **A** but for macaque hippocampal LFP. **C** Upper, saccade onset-triggered macaque hippocampal LFP. Lower, theta power (normalized) as a function of saccade magnitude in the macaque hippocampus. Norm., normalized. **D** Left, LFP power from 3 Hz to 12 Hz in the human hippocampus during target search and no target search tasks. Middle, LFP power from 3 Hz to 12 Hz at the boundary and away from the boundary (inner) during both tasks. Right, LFP power across room locations. **Ea** Upper, firing rate map of a human hippocampal neuron during virtual navigation; lower, the corresponding LFP phase as a function of position near the peak firing location (dashed circle in the upper panel). **Eb** Spike-LFP phase autocorrelogram of an example neuron in the macaque hippocampus during free foraging, showing gradual theta phase precession. **Aa** and **Ab** were generated using a public dataset [63]. **Ac** was adapted with permission from [64]. **B**, **C**, and **Eb** were adapted with permission from [35]. **D** was adapted with permission from [60]. **Ea** was adapted with permission from [65].

Hippocampal local field potentials (LFPs) show prominent theta band activity (5 Hz–10 Hz) whenever rodents are in a moderately attentive state, including locomotion (Fig. 1A). Theta frequency and power increase as the speed of locomotion increases [39, 40]. Theta oscillations can arise from the interplay of multiple intrinsic and extrinsic factors, including GABAergic and cholinergic projections from the medial septum, and theta-rhythmic membrane resonance, among others [41, 42]. However, the precise mechanisms remain unclear. Neuronal activity exhibits an intricate relationship with theta, namely phase-locking and phase-precession. Different types of neurons show a preference for spiking at different theta phases [43]. Hippocampal place cells show progressive theta phase advancement as the animal passes the place fields (known as phase-precession) [44], forming theta sequences that may strengthen the already-existing (weak) synaptic connections among neurons within an assembly [45, 46]. Such a mechanism could facilitate asymmetric coupling from place cells activated earlier in a theta cycle to those later in the cycle. As a consequence, later place cells are activated by earlier place cells, causing a shift of the place field toward the direction from which the animal is coming (in a linear track) [47]. This experience-dependent asymmetric expansion of place fields (asymmetric excitation), when combined with a periodic inhibitory input (presumably at theta rhythm), in return contributes to the generation of theta sequences [48]. Theta sequences are also linked to trajectory planning [49] and are reactivated at a faster pace during sleep [50]. Theta rhythm/sequence abolition by medial septum inactivation impairs performance in a spatial alternation task [51]. Disruption of awake theta sequences while preserving behavioral timescale sequences disrupts sleep replay [52]. These results suggest that theta sequences underlie memory formation.

Despite the prevalence of theta oscillations in the hippocampus of rats and mice, they do not generalize across mammalian species. The bat hippocampus shows only intermittent theta bouts both during crawling and free flight, suggesting a lack of effect of locomotor speed [20, 25]. But what these short theta bouts correlate with has not been explicitly studied so far. It is possible that theta bouts correlate with transient behavior such as the onset of locomotion and saccadic eye movement. Or alternatively, they are correlated with the main sensing mode or its specific attributes during exploration—whisking and sniffing in rodents and echoing in bats [53]. Besides, bat hippocampal neuronal activity shows little theta rhythmicity, despite rodent-like place coding patterns. Nevertheless, neurons show non-rhythmic phase-locking and precession relative to LFPs of a broader frequency, 1 Hz–20 Hz [54]. These results suggest a decoupling between theta oscillations and phase-coding, which may have important implications for computational models of phase-precession [44, 48, 55].

## Human Hippocampus Shows Mixed Low and High Theta Activity

Results about theta activity obtained from primates, including humans, have been mixed. The exact range of the theta band also varies across studies. Earlier human studies have mostly used virtual reality assays. These studies have consistently found low-frequency theta oscillations (1 Hz–4 Hz) during virtual navigation [32, 56]. Regular theta and low theta indistinguishably show modulation by movement speed and other task variables during virtual navigation [31, 57]. Theta power is generally significantly larger during movement than during stillness. It is also associated with movement onset and with the length of the path to be taken [58], suggesting a role in path integration. The dominant theta frequency can shift in a task-dependent manner. Consistent with rodent studies, theta generally reflects global activity across the hippocampus and neocortex, suggesting its role in coordinating brain-wide activity during task execution.

A recent study extended previous results to freely-moving humans [59]. This confirmed two major findings: (1) theta occurs in short bouts; and (2) theta is more prevalent during fast *versus* slow motion. More intriguingly, it showed stronger theta activity in a congenitally blind patient than in sighted participants. This may reflect that, when vision is unavailable, we may adaptively shift to rely more on internal maps for navigation, which posits different demands on hippocampal involvement. One apparent limitation of this study is that the analog bandpass filter has a low end of 4 Hz, the frequency under which was thus not sufficiently sampled, masking the possibility that low theta activity is more prevalent than regular theta.

Large variability across participants is evident in human studies. Some participants show local maxima in the power spectrum at 7 Hz–9 Hz while others at 2 Hz–4 Hz, during either memory-based navigation or random walking in the real or virtual world [30]. Differences in the exact subregion and layer of the recording site may have contributed to the variability. An alternative explanation is that human theta is indeed more variable across participants, perhaps due to its relationship with many facets of ongoing cognitive processing, including internal, spontaneous activity. This non-trivial variability may have contributed to previous seemingly inconsistent findings. Overall, stopping appears to reduce the low-frequency power, although the effect is often small with marginal significance. The role of attention on theta activity is not to be neglected. Theta band activity in freely-ambulating humans is modulated by the participant’s proximity to environmental boundaries [60]. Memory demand is also positively correlated with theta power [30, 60]. When participants need to actively search for a target, hippocampal theta power increases, compared to a “no search” task (Fig. 1D). This effect may be aligned with the influence of attention. Similarly, higher frequency theta activity may be associated with tasks that require higher spatial attention but is not due to a difference between real *versus* virtual navigation as suggested by a few studies [30, 59, 61]. Indeed, higher theta is more prominent when participants need to memorize a fixed location in the environment to drop off passengers, which would require a higher level of attention than finding randomly placed passengers [31].

Another study used a different virtual spatial memory task in which the participant’s speed in the virtual track varied randomly, therefore requiring the participants to pay more attention to their location during movement [62]. This excludes the possibility in previous studies that participants could estimate their location by time if speed were held constant. This shows that both low and regular theta exist along the entire hippocampal long axis, the posterior part showing a slightly higher proportion of regular theta. The posterior hippocampus also contains a larger fraction of electrodes that show a significant correlation with movement speed, for both low theta and regular theta. It is therefore hypothesized that the posterior hippocampus is more involved in spatial processing whereas the anterior part is more generally involved in broader functions including episodic memory. However, it is by no means that low and regular theta reflect a clear-cut border rather than a gradient along the hippocampal long axis. A thorough investigation requires an even distribution of electrodes along the entire long axis, and within the same subregion.

## Eye Movements Modulate Theta Activity in Non-human Primates

Earlier studies have identified theta rhythms (7 Hz–9 Hz) in anesthetized squirrel monkeys [66]. Two key findings have been confirmed in later studies that suggest an important departure from rodents: one is the co-existence of considerable low-frequency theta activity, the other is that it has a much shorter duration. Later studies have also extended the experimental paradigm to awake, behaving animals. In freely-moving marmosets in a linear track, theta oscillations occur only in sporadic bouts rather than continuously, and there is a limited spike-theta relationship for place coding [33]. Similarly, macaque hippocampal theta is also short-lived with a peak power below 5 Hz, lower than that found in marmosets, closer to what has been found in some human studies [35, 67] (Fig. 1B). Theta frequency increases with speed, similar to findings in rodents, albeit only with slow motion and remains mostly steady at speeds larger than ~30 cm/s; theta frequency also increases with acceleration (Fig. 1B). The disentanglement of the two (speed and acceleration) calls for future more controlled experiments.

The bulk of studies has linked hippocampal theta activity to eye movements in macaques. Saccadic eye movements evoke robust field potentials in the theta band locally in the hippocampus, even in darkness, suggesting an extra-retinal, likely motion-related source [68]. This is analogous to the sniffing- and vibrissa-locked theta reported in rodents [53]. In this regard, primates and rodents are similar (synchronization between the main sensorimotor process and field potentials in the hippocampus). Saccades also realign the hippocampal theta phase [35, 69, 70], which may provide a temporal window for focused information processing during active sensing. Moreover, pre-stimulus theta power correlates with later recognition performance [70]. Saccade magnitude is linearly correlated with theta power in the hippocampus [35, 71] (Fig. 1C). These results reveal a tight relationship of hippocampal theta timing and magnitude with information encoding and memory. These results, combined with rodent studies, establish a firm link between theta and exploratory behavior. After all, the behavioral correlates of theta activity may not be that different across mammalian species.

It has been proposed that the hippocampus may receive a corollary discharge of saccade motion, which may underlie predictive coding. This is supported by the finding that saccade modulation occurs before its onset (Fig. 1C). Saccades also evoke robust potentials in regions other than the hippocampus and medial temporal regions, including the medial septum and prefrontal cortex. Therefore, it is a reasonable hypothesis that saccades synchronize brain-wide activity in preparation for upcoming fixation to maximize information gathering.

## Theta Phase Coding in Primates

As discussed above, spike timing of hippocampal neurons may be important for neural plasticity and sequence learning, and spikes from different neurons can be coordinated by fluctuations in LFPs. Such temporal coordination among neurons within an assembly may underlie the mechanisms of memory and other cognitive processing, linking sequential events to form a coherent representation. Despite the clear differences in theta activity between primates and rodents, phase-coding appears to be conserved, at least to some extent, across mammalian species.

First, human hippocampal neurons are phase-locked to low theta activity, showing preference at various phases [72]. Stronger phase-locking predicts more successful memory formation [73]. This supports the hypothesis that synaptic plasticity associated with theta phase-locking underlies memory-related behaviors. Moreover, neuronal firing in the hippocampus is locked to the theta phase that is correlated with upcoming navigational goals, without significant changes in firing rate [74]. In freely-moving macaques, hippocampal neurons are tuned to various oscillations in different bands. The vast majority of neurons show modulation by low theta activity, and spatially-tuned neurons show stronger phase-locking than neurons without spatial selectivity [35]. Thus, like rodents, theta phase-locking in primates may be an important form of neural code for higher cognitive processing, including memory and navigation [75, 76].

Another aspect of theta phase-coding pertains to phase-precession. Human hippocampal neurons exhibit theta phase-precession as a function of location or progression toward specific goals (Fig. 1E), although there is a lack of rodent-like place coding [65]. The goal-related phase-precession is interesting, for it extends spatial phase-precession in rodents to a more general regime in humans. A rather low fraction of hippocampal neurons in both marmosets and macaques show theta phase-precession during free movement [33, 35] (Fig. 1E). One thing to note here is that, in the absence of a rodent-like place code, one can analyze phase-precession by comparing the frequency component of the spike phase autocorrelogram and non-oscillatory LFP [77].

## Spatial Code in Rodents and Bats

Originally discovered in the rat hippocampus, place cells are perhaps one of the most striking examples of a “simple” correspondence between an internal neural code and the complex external environment. Place cells fire at specific locations, forming a precise population representation of the entire space the animals occupy [17]. The place-code is sparse (a given cell fires at one or only a few locations) and orthogonal (population activity is independent at different locations). A place-code can arise from and be modulated by the interplay among multiple factors, including self-motion, distal landmarks, local cues (including reward), trajectory planning, and likely many more. Lesion and single-cell manipulation experiments have established the causal role of the hippocampus in spatial cognition [7, 21]. In the adjacent entorhinal cortex, some cells have multiple fields that are organized in a periodic hexagonal pattern [23], therefore named grid cells. First found in the rat subicular complex, head direction cells show sharp tuning to the allocentric direction the animal faces [24]. These functional cell types have also been identified in the bat hippocampal formation, and they extend to three dimensions, although the exact properties differ from representations in two dimensions [19, 20, 25–28].

Since the discovery of place cells, it has been a long-standing question whether there is an analogous place-coding scheme in primates. Most studies along this line have been carried out during a virtual-navigation task in head- and body-restrained primates, including humans. Only a few studies have examined hippocampal response properties in partially or fully freely-moving monkeys. We discuss these findings below.

## Various Spatial Signals in Primates Under Restraint

**Fig. 2:**
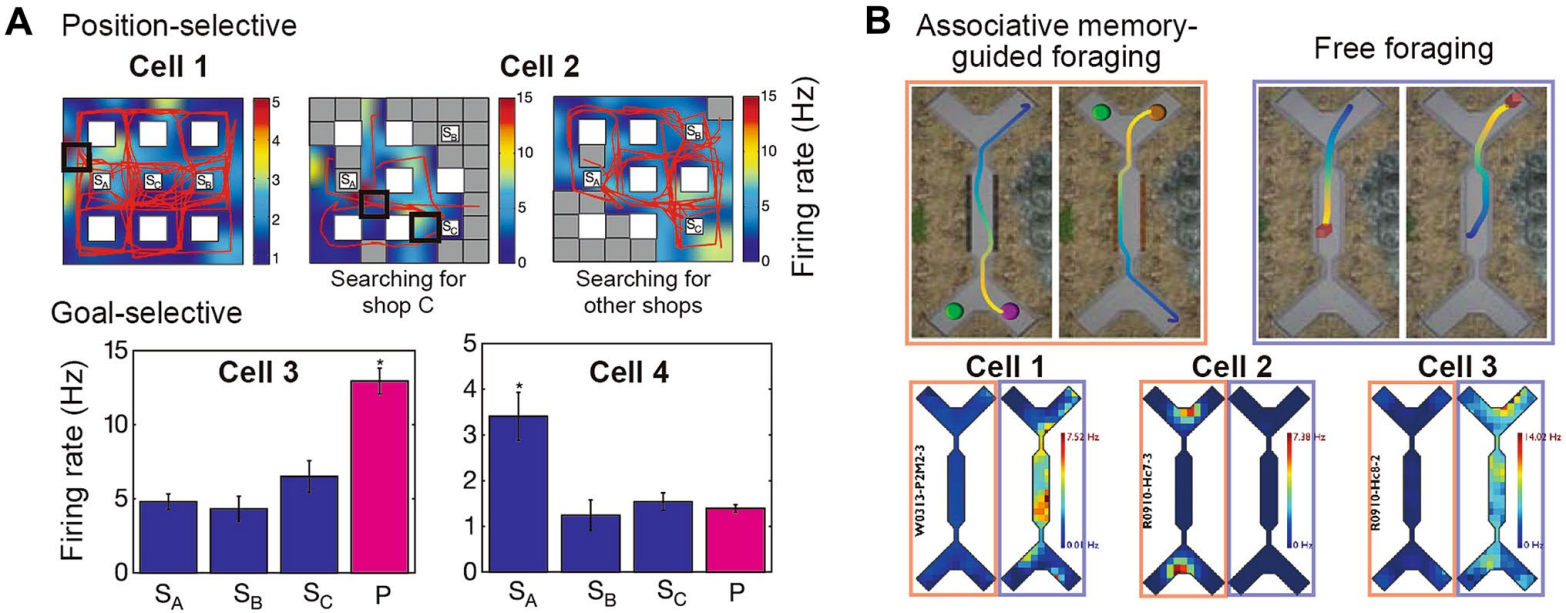
Task- and goal-dependent spatial responses in the primate hippocampus. **A** Upper, position-selective responses of two example neurons in the human hippocampus; cell 2 is position-selective only when searching for shop C (S_C_) but not other shops (S_A_ and S_B_). Note the firing is more distributed than typical rodent place cells. Lower, firing rate histograms of two example goal-selective neurons when searching for different goals (P, passengers). **B** Upper, macaque monkeys are trained to perform an associative memory-guided foraging task (choose different targets depending on the material along the corridor) and a free-foraging task in the same spatial environment. Lower, firing rate maps of three example hippocampal neurons showing distinct activity patterns between the two tasks. **A** is adapted with permission from [34]. **B** is adapted with permission from [91].

Intracranial recordings in human patients have identified hippocampal neurons selective to locations in virtual reality, and also to navigational goals and often to the conjunction between variables [34] (Fig. 2A). Neurons with selectivity to virtual heading direction, spatial goals, and task progression have also been identified across human medial temporal lobe regions [78]. Like rodents, egocentric tuning (relative to a virtual body reference frame) is also present in the parahippocampal cortex [79]. This type of experiment, while proven to be useful to bridge the knowledge gap between rodents and humans, can fall short when translated into real-world situations. The main reason is that the subjects predominantly use visual cues (visual landmarks and optic flow) alone to move about in the virtual environment with a near absence of vestibular, proprioceptive, and motor inputs. This brings about sensory conflict, or a change of gain between sensory modalities, which could alter spatial representations in an unpredictable way. We know from rodent studies that clamping vestibular inputs disrupts or rescales place fields and changes theta oscillations [80–82]. Therefore, despite the fruitful findings about neural correlates of space in the human brain, the interpretation of these results awaits careful consideration due to the apparent limitations [83].

Like human studies, monkey studies have mostly used head-fixed preparations with a handful of exceptions. Perhaps unsurprisingly, these studies focus on looking for neural correlates of virtual space and other spatial variables during virtual navigation. Hippocampal neurons have been shown to be correlated with various spatial variables, including stimulus location, self-position, head direction, and attended location. When sitting head-fixed in a chair in front of a monitor, some neurons in the macaque hippocampus show selectivity for objects, view location, or the conjunction between the object (or reward) and location (place in a scene) [84–89], which might be important for associative memory. When switched to a virtual-navigation task, many more hippocampal neurons become spatially responsive, the scale of which grows proportionally with the size of the virtual space; whereas a lower fraction of parahippocampal neurons shows spatial selectivity but more to virtual landmarks [90]. Such “spatial” responses could reflect the encoding of a combination of sensory, mnemonic, and spatial information. When visual cues are changed, population activity changes in a way predicted by visual features in the current and previous trials, suggesting a role of sensory input history (or memory) [91]. Coding of virtual space of the same environment also strongly depends on the task, be it a free foraging or a memory-dependent task [91] (Fig. 2B).

During a goal-oriented virtual-navigation task in a 5-arm maze, head-fixed macaques must rely on distal landmarks to infer the hidden goal location—so this is more likely to be a hippocampus-dependent task. Unlike rodent place cells, hippocampal neurons carry position information along a trajectory in a complex way. Many of these cells also encode the specific landmark being viewed and the bearing to the landmark [38]. Therefore, when mapping hippocampal activity onto either position, direction, or point of gaze, the tuning curves typically look “noisy”. These results suggest that the primate hippocampal code is complex and multidimensional, at least in the present task, likely reflecting the various elements embodied in a memory.

Entorhinal grid coding of self-location is even more scarcely investigated by direct recordings of single neurons in primates. The primate entorhinal cortex is not as easily accessible as the hippocampus, given its smaller size and more ventral-medial location. In human epileptic patients, rare opportunities for intracranial recordings from the entorhinal cortex reveal a grid-like code for virtual position [92, 93]. Functional magnetic resonance imaging experiments also reveal grid-like, 6-fold symmetry in blood-oxygen-level-dependent signals as a function of virtual movement direction [94]. However, these grid-like firing maps are noisier than rodent grid cells, and they are less frequently encountered. Moreover, human experiments usually involve memorizing specific locations and then performing memory-guided navigation, rather than the free-foraging paradigm used in rodent studies. It is possible, however, that memory-guided navigation in primates is analogous to free foraging in rodents, in terms of cognitive demands. Rodent grid cell firing in a 2D environment depends on head direction inputs [95]. It is likely that the reported grid codes in humans and rodents differentially rely on self-motion, and therefore may involve distinct mechanisms. Without further corroborative studies, particularly in freely-moving primates, it remains difficult to draw a direct comparison between species. Indeed, one study has shown that there is little grid firing in the entorhinal cortex of freely-foraging macaques [35]. Whether a grid code would emerge in a more cognitively demanding task and how it is modulated by other behavior-relevant variables calls for future studies along the same line.

## Place Selectivity in an Open Environment in the Monkey Hippocampus

In another series of studies, macaques were trained to drive a cart using a joystick in an open environment [96–98]. Hippocampal neurons showed increased activity at specific locations, reminiscent of rodent place cells, though the peak firing rates were generally much lower, and the firing fields were less sparse (lower signal-to-noise ratio). A potential caveat here is that the cart was usually moved at very low speed and low acceleration, which may not necessarily trigger navigationally meaningful vestibular responses. Addressing these caveats, monkeys were placed in a test chair that was precisely controlled by a robot. Moving at a speed and acceleration surpassing the vestibular thresholds, some hippocampal neurons were tuned to linear translation and axial rotation but much less so to self-location [99]. These responses can be explained by a combination of vestibular and visual inputs. A much lower percentage of hippocampal neurons respond in a setting with lower cognitive demands, e.g. when remaining at a fixed location or when moved passively by an experimenter [97]. These results again suggest that hippocampal activity strongly depends on the specific task and concurrent cognitive demands.

Under truly unrestrained conditions, elevated firing at specific locations has been reported in the monkey hippocampus [33, 35, 100, 101]. The place responses recorded diverge in several regards from the counterpart in rodents. First, monkey hippocampal neurons almost always show mixed responses, encoding position, head direction, task, and many other behavior-relevant aspects. Second, the proportion of place-responsive neurons is much lower in monkeys. Last, monkey “place fields” are often dispersed and not as sparse, and the peak firing rate is much lower than that in rodents. Therefore, place itself does not appear to be the primary driver of firing in the monkey hippocampus. That said, the task-dependence of hippocampal responses may explain the low place selectivity reported so far. Free foraging *per se* may entail distinct navigation strategies in rodents and primates, that is, place navigation and beacon navigation (viewpoint-dependent), respectively. Growing place awareness can be brought about by increasing the task complexity, for example by using a dry land version of the Morris water maze task. It is also likely that room size or complexity affects hippocampal place responses. In a compartmentalized environment where monkeys are unable to see through but must navigate around to see what happens where, place selectivity may be increased [102]. This also suggests that when trajectories need to be learned, hippocampal spatial responses may be increased. In such segmented environments, it would be interesting to determine how the hippocampal spatial code remaps across subspaces, when one subspace is or is not directly visible from other subspaces [103].

The above primate studies have almost exclusively investigated hippocampal place responses in two-dimensional (2D) environments; three-dimensional (3D) properties are largely unknown. Many monkey species are arboreal, so whether extending the analysis to the third dimension would expand spatial responses remains to be seen. Initial results suggest that this is the case [101]. Nevertheless, a direct comparison between 2D and 3D place representations is needed.

Despite the major differences in their spatial codes between primates and rodents, the firing statistics of hippocampal neurons appear to be consistent [96, 104, 105]. The firing rate distributions show a log-normal pattern, with the majority firing at a very low rate. The average firing rate is ~1 Hz. Such sparse activity is not trivial. It may reflect the unique computation performed by the hippocampal circuit, for example, to orthogonalize similar inputs and to increase storage capacity. This property could be the common core algorithm of the hippocampus across species, and it could bridge the gap in our understanding of hippocampal functions.

Overall, neuronal correlates of place in the primate hippocampal formation are multiplexed and highly dependent on specific tasks. To fully explore the potential of the hippocampal response regime, future studies would require simultaneous recordings from many neurons across moderately complex tasks under ethological conditions. The resulting datasets will undoubtedly bring the complexity to another level, which will require the development of novel methodologies of data analysis. For example, recent applications of multimodal model-based approaches have revealed that neurons in many regions encode more features than previously thought [35, 106, 107], but only when all accessible variables are considered simultaneously.

## Eye Movement Coding and Head Versus Eye Reference Frames

**Fig. 3:**
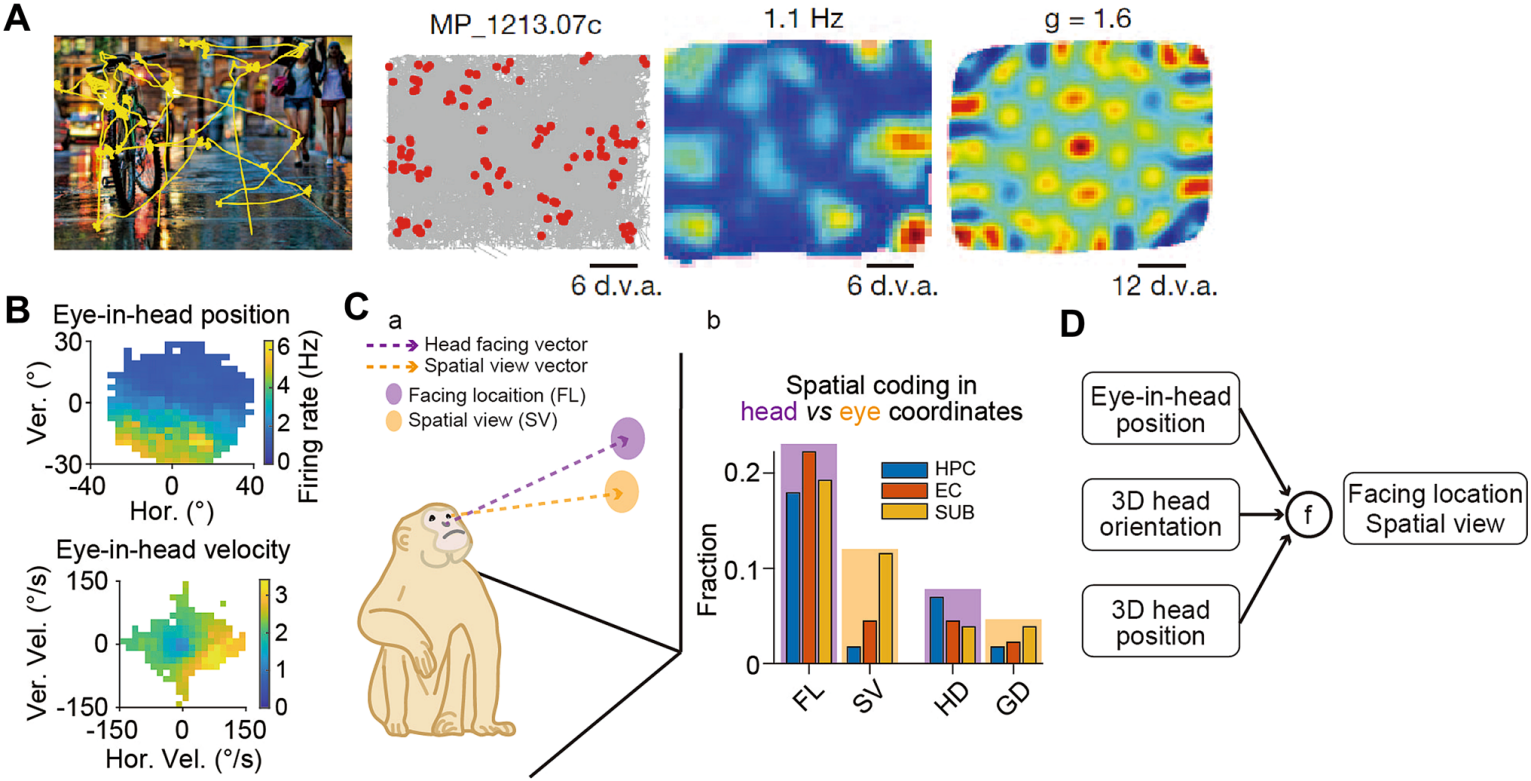
Visual spatial responses and head *versus* eye reference frames. **A** An example neuron from the entorhinal cortex showing grid-like firing maps when the head-fixed macaque is freely viewing static pictures. From left to right: eye scan path on an example picture, spikes overlaid on eye movement trajectories [in degrees of visual angle (d.v.a)], color map of the neuronal firing (peak firing rate is indicated at the top), and autocorrelation of the firing map to the left [grid score (*g*) is indicated at the top]. **B** Firing rate maps of two example neurons as a function of eye-in-head position and velocity (Vel.) in freely-moving macaques. Ver., vertical; Hor., horizontal. **Ca** Diagram showing head facing location and eye spatial view. **Cb** Fraction of neurons encoding facing location (FL), spatial view (SV), horizontal head direction (HD), and gaze direction (GD), across hippocampal regions (HPC, hippocampus; EC, entorhinal cortex; SUB, subicular complex). **D** Simplified schematic illustrating that integration of egocentric eye-in-head, allocentric 3D head orientation, and position gives rise to allocentric facing location and spatial view information. **A** is adapted with permission from [37]. **B** and **Cb** are adapted with permission from [35].

Primates primarily rely on vision and internal maps to navigate. A rather fruitful line of research focuses on eye movement-related signals in the primate hippocampal formation. About 20% of hippocampal neurons selectively respond to view locations on a screen [108]. A similar proportion of entorhinal cortex neurons show visual spatial responses (grid and border) [37, 109] (Fig. 3A). A recent study extends this result to freely-behaving macaques and further shows hippocampal tuning to eye-in-head velocity (including saccadic eye movement) [35] (Fig. 3B). However, grid tuning in the visual space turns out to be much weaker than the rodent grid code. This raises the possibility that the primate grid code is different from that of rodents, although it remains to be determined if the neurons are from the subregion homologous to the dorsal-medial portion of the superficial entorhinal cortex in rats. In a goal-directed virtual-navigation task, macaques adaptively use their gaze to guide movement even after the transient appearance of the goal at a distance [110]. Likewise, human participants actively make rapid eye movements to interrogate the environment while keeping the goal location in their mind’s eye (i.e., the goal is now at this gaze location) [111, 112]. Eye movement thus is a meaningful window in which to investigate trajectory planning and sequential decision-making during navigation. Forward sweeps from the current location to the goal location manifest possible behavioral correspondence of the neural pre-play reported in rodents during planning [113]. Further, gaze toward other locations could indicate subjects’ uncertainty (or deliberation) about the goal location. Future studies are needed to bridge the gap between such eye movements and their neural correspondence.

The above experiments were mostly carried out in stationary subjects, and it is currently unknown how these results can be extrapolated into naturalistic conditions. Eye movement patterns are likely different during free behavior from those during virtual navigation. During free behavior, we actively rotate our heads to face a certain direction and use our gaze to explore the details around that direction. This could link the implications of the hippocampus in spatial exploration and visual memory. Compared to rapid eye movement, head orientation appears to be a more stable anchor for firing fields to be attached to. Moreover, macaques use different strategies to make decisions in the real world: They are more risk-aversive during natural behavior while more risk-seeking under restrained conditions [114]. Given the link between decision-making and eye movements during virtual navigation, it remains to be seen if such a change in decision strategies is reflected in subtle gaze behavior.

Rodent studies have consistently failed to disambiguate head direction and gaze direction tuning. It is still unclear whether head direction tuning reflects gaze direction. Earlier studies by Rolls and colleagues using macaques in a cart (the head is held in a forward-looking position) have identified hippocampal neurons tuned to where the animal looks, i.e., spatial view cells [36, 115]. These neurons are tuned to “visual space” but not to the animal’s location. More cells become selective to spatial view in a visually richer environment [116]. Some cells maintain the tuning when the view details are obscured, and some continue firing when that location is being viewed even in darkness when the head is steady [117, 118], suggesting a role in memorizing what and where something is seen at a distance. It should be noted that the overall responses are complex in that some cells lose their tuning when visual inputs are obscured. Although the animals could turn rapidly, the caveats in these studies include that the monkey’s head is held in an upright position and the peak translational velocity is relatively low at 0.6 m/s [115], masking the possibility that natural head motion would make the story more complex.

Indeed, when the monkey’s head is free to move in 6 degrees of freedom, facing location (where the head points) coding outperforms spatial view [35] (Fig. 3C). The same holds true for azimuth directional tuning. It remains to be seen if increasing visual richness of the environment would reverse this conclusion. Unlike in head-fixed animals, there is a near absence of grid tuning for where the monkey looks or where the head points in freely-behaving animals. Future studies need to examine what these visual grid cells do during free navigation. To compute spatial view or head-facing location, one needs to integrate a mixture of egocentric and allocentric coordinates, including eye-in-head position, head orientation, and location in the three-dimensional allocentric space (Fig. 3D). Such a transformation may involve many brain regions, of which the retrosplenial/precuneus complex may be at the core [119, 120], given its role in integrating internal hippocampal and external visual information [121, 122]. However, encoding of different reference frames may manifest as a continuum rather than a clear cut along the medial temporal lobe-retrosplenial/precuneus-parietal circuits.

Indeed, there is strong head-tilt tuning across hippocampal regions, similar to that reported in the mouse limbic system, the bat presubiculum, and the monkey anterior thalamus [26, 123, 124]. These results suggest that gravity is an important reference to anchor orientation tuning. Combining 3D head orientation and location, with environmental geometry, one can obtain facing location (where the head points, i.e., the intersection between head pointing vector and environmental boundaries) (Fig. 3C). Hippocampal neurons are consistently best explained by this variable. We note here it remains to be explored what facing location truly means. For example, it is possible that facing location reflects egocentric directional tuning relative to certain landmarks in the environment, similar to what has been shown in the rodent hippocampal system [125]. Similarly, future analyses need to assess whether the rodent hippocampus encodes facing location.

There are still a few missing pieces when translating between egocentric and allocentric reference frames. One of them is the differentiation between head orientation in the allocentric frame and head orientation with respect to the body. No study so far has differentiated the two reference frames in the hippocampal formation. There are some hints from rodent studies that the head-trunk relationship is encoded in the posterior parietal cortex [126]. To capture the full repertoire of primate behavior in an unrestrained condition, one needs to combine portable eye tracking and accurate tracking of the whole body (head, back, and limbs). Recent advances for markerless pose estimation are particularly helpful [127–130].

## Concluding Remarks and Open Questions

In this review, we have discussed progress towards understanding the hippocampal correlates of spatial behavior in primates at the level of single neurons and LFPs. There are some similarities but also marked differences between primates and rodents. The lower-frequency hippocampal theta in primates is intermittent and modulated by eye movements, whereas theta phase-coding is somewhat present. Hippocampal neurons often show mixed selectivity to various spatial variables, with head orientation and eye movement properties predominating over the place and grid code. The hippocampal spatial code is also highly task-dependent, manifesting the role of cognitive demands. Below, we discuss some open directions that may be interesting to pursue.

Regardless of what the hippocampal code is, it needs to be read and used by downstream regions, which often have topographical connections with the hippocampus. For example, the retrosplenial cortex receives strong projections from the posterior hippocampus while the orbitofrontal cortex mostly connects with the anterior portion [131, 132]. The hippocampus is also part of the Papez circuit and the default mode network [133, 134]. Understanding how networks of regions (centered on the hippocampus) communicate during online and offline processing is key to gaining a firmer grasp of the brain-wide mechanisms of spatial cognition and memory. The macaque model offers a unique opportunity to investigate this question at a high spatiotemporal resolution on a large scale.

The anatomy of the macaque hippocampus embraces two key aspects that are distinct from rodents: first, the anterior portion of the hippocampus is disproportionally larger than the posterior part; and second, the hippocampi in the left and right hemispheres share much fewer direct connections than rodents [135]. Such anatomical differences may likely underlie more significant functional differentiations in the macaque hippocampus. Future studies need to examine simultaneous neural activity along the entire long axis and across both hemispheres. That said, the hippocampus is an evolutionarily old structure. There are good reasons to believe that the basic algorithms are preserved across mammalian species. However, such preservation may not be shown as a task-dependent spatial code at the single neuron level. It would be useful to determine how population activity patterns (ensemble dynamics) are similar or different across tasks, behavioral states, and even across species. In this way, we would better understand how the hippocampal circuit transforms its inputs to its outputs. This would require recordings from many neurons across many regions under different situations with multiple spatial and temporal scales.

With the emergence of advanced techniques for monitoring the rich behavior of freely-behaving non-human primates, when combined with large-scale telemetric recordings and sophisticated tasks, the time is ripe for investigating the role of the hippocampal-neocortical networks in spatial cognition and other higher functions under moderately ethological conditions.
